# Expression of PD-L1 on Canine Tumor Cells and Enhancement of IFN-γ Production from Tumor-Infiltrating Cells by PD-L1 Blockade

**DOI:** 10.1371/journal.pone.0098415

**Published:** 2014-06-10

**Authors:** Naoya Maekawa, Satoru Konnai, Ryoyo Ikebuchi, Tomohiro Okagawa, Mami Adachi, Satoshi Takagi, Yumiko Kagawa, Chie Nakajima, Yasuhiko Suzuki, Shiro Murata, Kazuhiko Ohashi

**Affiliations:** 1 Department of Disease Control, Graduate School of Veterinary Medicine, Hokkaido University, Sapporo, Japan; 2 Veterinary Teaching Hospital, Graduate School of Veterinary Medicine, Hokkaido University, Sapporo, Japan; 3 North Lab, Sapporo, Japan; 4 Research Center for Zoonosis Control, Hokkaido University, Sapporo, Japan; Mie University Graduate School of Medicine, Japan

## Abstract

Programmed death 1 (PD-1), an immunoinhibitory receptor, and programmed death ligand 1 (PD-L1), its ligand, together induce the “exhausted” status in antigen-specific lymphocytes and are thus involved in the immune evasion of tumor cells. In this study, canine PD-1 and PD-L1 were molecularly characterized, and their potential as therapeutic targets for canine tumors was discussed. The canine PD-1 and PD-L1 genes were conserved among canine breeds. Based on the sequence information obtained, the recombinant canine PD-1 and PD-L1 proteins were constructed; they were confirmed to bind each other. Antibovine PD-L1 monoclonal antibody effectively blocked the binding of recombinant PD-1 with PD-L1–expressing cells in a dose-dependent manner. Canine melanoma, mastocytoma, renal cell carcinoma, and other types of tumors examined expressed PD-L1, whereas some did not. Interestingly, anti-PD-L1 antibody treatment enhanced IFN-γ production from tumor-infiltrating cells. These results showed that the canine PD-1/PD-L1 pathway is also associated with T-cell exhaustion in canine tumors and that its blockade with antibody could be a new therapeutic strategy for canine tumors. Further investigations are needed to confirm the ability of anti-PD-L1 antibody to reactivate canine antitumor immunity *in vivo*, and its therapeutic potential has to be further discussed.

## Introduction

Recently, cancer has been a major cause of death in dogs and has surpassed infectious diseases. According to a recent report, 27% of overall dog deaths is due to cancer [1]. Current clinical approaches for canine cancer are surgical, chemotherapeutic, and radiation therapies, as in humans. In some cases of dog cancers, however, it is difficult to treat them by only using existing therapeutic methods because of the severe stress, adverse effect, and/or difficulties in approaching the tumor sites. In addition, the sensitivities to the chemo/radiotherapies can differ dependent on the tumor types. Therefore, it is worth investigating the efficacy of novel approaches against canine cancer, including immunotherapy, as this may lead to the development of more effective therapies that can induce tumor remission.

Programmed death 1 (PD-1), an immunoinhibitory receptor, and programmed death ligand 1 (PD-L1), its ligand, together can induce the “exhausted” status in antigen-specific lymphocytes and are thus involved in the immune evasion of tumor cells [2], [3]. In human cancer, tumor cells express PD-L1, and the cells cause T-cell dysfunction in breast cancer [4], pancreatic cancer [5], [6], and bladder cancer [7]. Tumor-infiltrating lymphocytes, which are specific to tumor antigens, express PD-1 in melanoma [8], lung cancer [9], and intrahepatic bile duct cancer [10]. Furthermore, in cases of renal cell carcinoma [11] and gastric carcinoma [12], patients with tumor PD-L1 are at a significantly increased risk of rapid disease progression and overall mortality, suggesting that PD-L1 is associated with poor prognosis in patients with tumors. When PD-1 binds to PD-L1, negative signals are transmitted into the lymphocytes, resulting in the suppression of antigen-specific immune responses [13]–[15]. Most importantly, this suppressive status is reported to be reversible, and the blockade of the PD-1/PD-L1 pathway using anti-PD-L1 antibodies or other molecules can restore the function of exhausted lymphocytes [16], [17]. It is also reported that the blockade of the PD-1/PD-L1 pathway results in reactivation of antitumor immunity and in subsequent regression of some tumors in human clinical trials [18], [19]. Therefore, this therapeutic strategy may be promising for effective treatment of tumors. In the veterinary field, however, reports on the PD-1/PD-L1 pathway are few and its relevance to diseases is almost unknown.

Recent studies in our laboratory have revealed that immunoinhibitory molecules including PD-1/PD-L1 play critical roles in immune exhaustion and disease progression in case of bovine leukemia virus (BLV) infection [20]–[26]. We established the specific antibodies; blocking using the antibodies in the inhibitory pathway *in vitro* increased cytokine responses and enhanced immune cell function, leading to decrease in the viral load [20], [21]. Therefore, evaluation of inhibitory receptor expression kinetics is essential to improve the development of an effective immunotherapy that can induce antitumor responses in dogs. In this study, canine PD-1 and PD-L1 were molecularly characterized. Then, PD-L1 expression on canine tumors and the potential of the PD-1/PD-L1 pathway as a therapeutic target for treatment of dog tumors were assessed in the lab.

## Materials and Methods

### Canine Samples

Animal use throughout this study was approved by the Institutional Animal Care and Use Committee (the serial number of approval was #1039), Graduate School of Veterinary Medicine, Hokkaido University, which has been fully accredited by the Association for Assessment and Accreditation of Laboratory Animal Care International. Peripheral blood samples were obtained from healthy 5- or 8-year-old Beagles kept at the Experimental Animal Facility, Graduate School of Veterinary Medicine, Hokkaido University. Clinical samples were surgically collected from dogs with tumors at the Veterinary Teaching Hospital, Graduate School of Veterinary Medicine, Hokkaido University in 2012–2013. For immunohistochemical analysis, tumor specimens kept at NORTH LAB (Sapporo, Japan) were used.

### Cell Culture

Cos-7 cells (African Green Monkey SV40-transformed kidney fibroblast cell line) [27] were cultured in RPMI 1640 medium (Sigma-Aldrich, St. Louis, MO, USA) supplemented with 10% fetal calf serum (FCS) (Valley Biomedical, Winchester, VA, USA), 2 mM L-glutamine (Life Technologies, Carlsbad, CA, USA), 200 µg/mL streptomycin (Life Technologies), and 200 U/mL penicillin (Life Technologies) at 37°C and 5% CO_2_. Chinese hamster ovary-DG44 (CHO-DG44) cells were cultured in CD-DG44 medium (Life Technologies) containing GlutaMAX supplement (20 mL/L, Life Technologies) and 10% Pluronic F-68 (18 mL/L, Life Technologies) at 37°C and 5% CO_2_. The canine melanoma cell lines CMeC [28], LMeC [28], CMM-1 [29], and CMM-2 [29] were cultured in RPMI 1640 medium supplemented with 10% FCS, 2×10^−5^ M 2-mercaptoethanol, 2 mM L-glutamine, 200 µg/mL streptomycin, and 200 U/mL penicillin at 37°C and 5% CO_2_. The canine mastocytoma cell lines CM-MC [30] and CoMS [31] were cultured in RPMI 1640 medium supplemented with 10% FCS, 12 mM HEPES, 2 mg/mL NaHCO_3_, 2 mM L-glutamine, 200 µg/mL streptomycin, and 200 U/mL penicillin at 37°C and 5% CO_2_. The canine osteosarcoma cell lines POS [32] and HM-POS [33] were cultured in D-MEM (Life Technologies) containing 10% FCS, 2 mM L-glutamine, 200 µg/mL streptomycin, and 200 U/mL penicillin at 37°C and 5% CO_2_. To stimulate the cells, the canine tumor cell lines were treated with 100 ng/mL canine recombinant IFN-γ (Kingfisher Biotech, St. Paul, MN, USA) and cultured for 24 h. Canine peripheral blood mononuclear cells (PBMCs) were purified from heparinized blood samples by density gradient centrifugation on Percoll (GE Healthcare UK, Buckinghamshire, UK) and cultured in RPMI 1640 medium supplemented with 10% FCS, 2 mM L-glutamine, 200 µg/mL streptomycin, and 200 U/mL penicillin at 37°C and 5% CO_2_. Concanavalin A (ConA) (5 µg/mL, Sigma-Aldrich) or PMA (20 ng/mL, Sigma-Aldrich) and ionomycin (1 µg/mL, Sigma-Aldrich) were added to the medium to activate lymphocytes.

### Identification of Canine PD-1 and PD-L1 Genes

Total RNA was isolated from the Beagle and the Samoyed PBMCs stimulated with ConA for 4 h, white blood cells of the Labrador retriever, testis tissue of the Japanese Akita, and lung tissue of the Bernese mountain dog, using TRIzol reagent (Life Technologies) according to the manufacturer’s instructions. Residual genomic DNA was removed from the total RNA by DNase (Life Technologies) treatment. cDNA was synthesized from 1 µg of the total RNA using Moloney murine leukemia virus reverse transcriptase (Takara, Shiga, Japan) and oligo-dT primer, as recommended by the manufacturer. To amplify the inner sequences of canine PD-1 or PD-L1, canine PD-1– and PD-L1–specific primers were designed based on the putative canine PD-1 and PD-L1 mRNA sequence reported in the GenBank database (XM_543338 and XM_541302). Canine PD-1 and PD-L1 cDNA were amplified from Beagle cDNA by PCR using primers 5′-AGG ATG GCT CCT AGA CTC CC-3′ (PD-1 inner forward), 5′-AGA CGA TGG TGG CAT ACT CG-3′ (PD-1 inner reverse), 5′-ATG AGA ATG TTT AGT GTC TT-3′ (PD-L1 inner forward), and 5′-TTA TGT CTC TTC AAA TTG TAT ATC-3′ (PD-L1 inner reverse). The PCR cycling conditions were as follows: (1) initial denaturation at 94°C for 5 min, (2) 40 cycles of denaturation at 94°C for 1 min, annealing at 58°C (PD-1) or 50°C (PD-L1) for 1 min and extension at 72°C for 1 min 30 s, and (3) final extension at 72°C for 7 min. PCR amplicons were purified using the FastGene gel/PCR extraction kit (Nippon Genetics, Tokyo, Japan), cloned into the pGEM-T Easy vector (Promega, Madison, WI, USA), and sequenced using the CEQ8000 DNA analysis system (Beckman Coulter, Fullerton, CA, USA). 5′-RACE and 3′-RACE were then performed using the 5′-RACE system for rapid amplification of cDNA ends and 3′-RACE system for rapid amplification of cDNA ends (Life Technologies), respectively. The gene-specific primers for the canine PD-1/PD-L1 used for 5′-RACE were 5′-GTT GAT CTG TGT GTT G-3′ (PD-1) and 5′-TTT TAG ACA GAA AGT GA-3′ (PD-L1). The gene-specific primers for canine PD-1/PD-L1 used for 3′-RACE were 5′-CGG GAC TTC CAC ATG AGC AT-3′ (PD-1) and 5′-GAC CAG CTC TTC TTG GGG AA-3′ (PD-L1). Based on the sequences obtained, new primer sets were designed to amplify the full length of the canine PD-1 and PD-L1 cDNA. PCR was performed using primers 5′-GGG GGA GGC GAG CAG G-3′ (PD-1 ORF F), 5′-GAG TCG AGA GAG GAG AGC CAT GAG-3′ (PD-1 ORF R), 5′-GCC AGC AGG TCA CTT CAG AAC-3′ (PD-L1 ORF F), and 5′-GCT GAA CTC AAA CCA CAG GCC-3′ (PD-L1 ORF R) as described above, except that the annealing temperature used was 60°C. The resulting amplicons were sequenced as described. To confirm the polymorphisms of the PD-1 and PD-L1 genes among canine breeds, the blood samples of the Beagle, Samoyed and Labrador retriever, testis tissue of the Japanese Akita, and lung tissue of the Bernese mountain dog were collected and studied using ORF primer and each the cDNAs of each sample. The established sequences were aligned, and unrooted neighbor-joining trees were constructed using the Mega version 5 software [34], [35].

### Preparations of Canine PD-1– and PD-L1–Expressing Cells

To construct the enhanced green fluorescent protein (EGFP) fusion expression vectors, the ORF region of the canine PD-1 and canine PD-L1 cDNA that did not have the stop codons was amplified by PCR using the cDNA obtained from the Beagle PBMCs and gene-specific primers [5′-CCG CTC GAG ATG GGG AGC CGG CGG GGG CC-3′ (PD-1 F, containing an *Xho*I restriction site), 5′-CGC GGA TCC TGA GGG GCC ACA GGC CGG GTC-3′ (PD-1 R, containing a *Bam*HI restriction site), 5′-GAA GAT CTA TGA GAA TGT TTA GTG TC-3′ (PD-L1 F, containing a *Bgl*II restriction site), and 5′-GGA ATT CTG TCT CTT CAA ATT GTA TAT C-3′ (PD-L1 R, containing an *EcoR*I restriction site)]. The amplicons were then cloned into the multicloning site of the pEGFP-N2 vector (Clontech, Palo Alto, CA, USA). These vectors were named pEGFP-N2–cPD-1 and pEGFP-N2–cPD-L1, respectively. For transient expression, Cos-7 cells (5×10^4^/cm^2^) were transfected with 0.4 µg/cm^2^ of the expression vector pEGFP-N2–cPD-1 or pEGFP-N2–cPD-L1 or empty pEGFP-N2 vector as the mock, using Lipofectamine 2000 (Life Technologies) according to the manufacturer’s instructions. The cells were cultured at 37°C and 5% CO_2_ for 48 h and harvested using a cell scraper. To confirm the expression of fusion proteins, the cells were observed under a confocal microscope LSM700 (Carl Zeiss Microscopy, Jena, Germany), and the subcellular distributions of EGFP were determined.

### Preparations of Recombinant Canine PD-1– and PD-L1–Rabbit IgG Fc Fusion Proteins (cPD-1–Ig, cPD-L1–Ig)

For the construction of the cPD-1–Ig and cPD-L1–Ig expression vectors, cDNA sequences of cPD-1 or cPD-L1 encoding the putative extracellular region of those proteins were amplified by PCR using the cDNA obtained from the Beagle PBMCs and the gene-specific primers [5′-CGC GGC TAG CAT GGG GAG CCG GCG GGG GCC-3′ (PD-1 F, containing an *Nhe*I restriction site), 5′-CGC GGA TAT CCA GCC CCT GCA ACT GGC CGC-3′ (PD-1 R, containing an *EcoR*V restriction site), 5′-CGC GGC TAG CAT GAG AAT GTT TAG TGT CTT-3′ (PD-L1 F, containing an *Nhe*I restriction site), and 5′-CGC GGA TAT CAG TCC TCT CAC TTG CTG GAA-3′ (PD-L1 R, containing an *EcoR*V restriction site)]. The amplicons were then cloned into the multicloning site of the pCXN-2.1–rabbit IgG Fc vector (kindly provided by Dr T. Yokomizo, Juntendo University; modified) [36], [37]. These vectors were named pCXN2.1–Rabbit IgG Fc-cPD-1 or pCXN-2.1–rabbit IgG Fc-cPD-L1, respectively. For stable expression, 4×10^6^ CHO-DG44 cells were transfected with 2.5 µg pCXN-2.1–rabbit IgG Fc-cPD-1 or pCXN-2.1–rabbit IgG Fc-cPD-L1 using Lipofectamine LTX (Life Technologies), as recommended by the manufacturer. Forty-eight hours later, the cells were collected and resuspended in the supplemented CD-DG44 medium containing 800 µg/mL G418 (Enzo Life Science, Farmingdale, NY, USA). Stably expressing cells were cloned using the limiting concentration technique, and high expression cell lines were obtained. The cell culture supernatant of these cell lines was collected after 7 days of the shaking culture. The supernatant containing Ig fusion proteins was concentrated by ultrafiltration using Centricon Plus-70 (Merck Millipore, MA, USA) and the Ig fusion proteins were purified by Ab-Capcher Extra (Protenova, Kagawa, Japan). Buffers were exchanged with phosphate buffered saline (PBS) using PD MiniTrap G-25 (GE Healthcare UK). The concentrations of the Ig fusion proteins were evaluated using the rabbit IgG ELISA quantitation set (Bethyl Laboratories, Montgomery, TX, USA). To confirm the expression of these Ig fusion proteins, sodium dodecyl sulfate–polyacrylamide gel electrophoresis (SDS–PAGE) and Western blot analysis with the Immobilon-P transfer membrane (Merck Millipore) were performed as described elsewhere [21]. The membrane was incubated with Immobilon Western chemiluminescent HRP substrate (Merck Millipore) to visualize the signals and analyzed by a Fluor-S MultiImager (Bio-Rad Laboratories, CA, USA).

### Flow Cytometry

To analyze the binding of cPD-1 to cPD-L1, cells that expressed cPD-1–EGFP or cPD-L1–EGFP were incubated with 10 µg/mL of cPD-1–Ig or cPD-L1–Ig or rabbit IgG isotype control antibody at room temperature for 30 min. The cells were washed twice and then incubated with Alexa Fluor 647-conjugated goat antirabbit IgG (H+L) F(ab’)2 (Beckman Coulter) at room temperature for 30 min. The cells were washed twice and analyzed by FACS Verse (BD Biosciences, San Jose, CA, USA) and FACS Express 4 (De Novo Software, CA, USA).

To detect the canine PD-L1 expressed on the cell surfaces, flow cytometry was performed using rat antibovine PD-L1 monoclonal antibodies [6G7-E1; rat IgM (κ), 5A2-A1; rat IgG1 (κ), 4G12-C1; rat IgG2a (κ)], which were established in our laboratory [20]. In brief, 1×10^6^ cells were incubated with 10 µg/mL antibovine PD-L1 antibodies at room temperature for 30 min and washed twice, followed by incubation with allophycocyanin-conjugated goat antirat Ig antibody (Beckman Coulter) at room temperature for 30 min. The cells were washed twice and analyzed. Rat IgM (κ) isotype control (BD Biosciences), rat IgG1 (κ) isotype control (BD Biosciences), and rat IgG2a (κ) isotype control (BD Biosciences) were used as isotype-matched negative control antibodies. PBS containing 10% goat serum (Sigma-Aldrich) was used in all washing processes and dilutions of Ig fusion proteins or antibodies.

To confirm the blockade of canine PD-1/PD-L1 binding by anti-PD-L1 antibody, cPD-L1–EGFP-expressing Cos-7 cells (2×10^5^) were incubated with various concentrations (0.5, 1.0, 2.5, and 5.0 µg/mL) of antibovine PD-L1 monoclonal antibody 6G7-E1 at 37°C for 15 min prior to incubation with 1 µg/mL (final concentration) of cPD-1–Ig at 37°C for 30 min. The cells were washed twice, and cPD-1–Ig, which was bound to the cPD-L1–EGFP-expressing cells, was detected by flow cytometry, as described above. As a negative control antibody, rat IgM (κ) isotype control (BD Biosciences) was used.

### Analysis of PD-L1 Expression on Single Cells from Canine Tumors

To obtain single cells from solid tumors, freshly excised solid tumor tissues were cut into small pieces and treated with 2 mg/mL collagenase D (Roche Applied Science, Indianapolis, IN, USA) in RPMI 1640 medium supplemented with 10% FCS, 2 mM L-glutamine, 200 µg/mL streptomycin, and 200 U/mL penicillin at 37°C for up to 2 h. The single cell suspension was washed twice with PBS and cultured in supplemented RPMI 1640 medium at 37°C and 5% CO_2_ for 24 h; this restored the cell surface PD-L1 expression, which had been degraded by the collagenase. The cells were harvested and the PD-L1 expression was analyzed by flow cytometry, as described above, using the antibovine PD-L1 monoclonal antibody 4G12-C1. In flow cytometric analysis, the cells were plotted using forward-scattered light (FSC) and side-scattered light (SSC), and the cell population with a higher FSC than lymphocytes was gated as the tumor cell population if there was no other population except for the population of tumor-infiltrating lymphocytes. To exclude any dead cells, the cells were stained with 7-amino-actinomycin D (BD Biosciences), and the live cell population was analyzed for PD-L1 expression. The canine PBMCs were used as a negative control under the same conditions.

### Immunohistochemical Analysis of PD-L1 on Tumor Cells

Formalin-fixed and paraffin-embedded tumor tissues were cut into 4-µm-thick sections and dried on silane-coated slides. The dried sections were deparaffinized in xylene. Antigen retrieval was performed in citrate buffer (0.37 g/mL of citric acid and 2.4 g/mL of trisodium citrate dihydrate) by microwave heating for 10 min. Endogenous peroxidase activity was blocked by incubating the sections in methanol containing 0.3% hydrogen peroxide for 15 min. Primary antibody incubation was performed at room temperature for 30 min using antibovine PD-L1 monoclonal antibody 5A2-A1 (1.2 µg/mL) or rat IgG1 isotype-matched control antibody (Biolegend, San Diego, CA, USA). The sections that were washed twice with PBS were incubated with Histofine simple stain MAX PO (rat) (Nichirei, Tokyo, Japan) at room temperature for 30 min, and positive staining was visualized with 3-diaminobenzidine tetrahydrochloride (DAB). The sections were observed under an optical microscope.

### Blocking Assay Using Anti-PD-L1 Antibody

Single cell suspensions from fresh solid tumor tissues were obtained by collagenase treatment, as described, or by a mechanical method using the 100 µm cell strainer (Becton, Dickinson and Company, Franklin Lakes, NJ, USA). The suspensions were underlaid onto 1.055 g/mL Percoll separation solution. The tumor cells were separated by density gradient centrifugation at 800×*g* for 20 min, and the cell pellets were collected and resuspended in the supplemented RPMI 1640 medium. Then, the cell suspensions were again underlaid onto 1.077 g/mL Percoll separation solution and centrifuged. The tumor-infiltrating lymphocytes in the gradient interfaces were collected and washed with PBS three times. These lymphocytes were resuspended in the supplemented (10% FCS, 2 mM L-glutamine, 200 µg/mL streptomycin, and 200 U/mL penicillin) RPMI 1640 medium.

The tumor-infiltrating lymphocytes and PBMCs obtained from the healthy adult Beagles (2×10^6^/mL) were cultured with 20 µg/mL of antibovine PD-L1 monoclonal antibody 6G7-E1 at 37°C and 5% CO_2_ for 48 h, and the culture supernatant was collected. As a negative control, low-endotoxin, Azide-free rat IgM isotype control antibody (Acris Antibodies, Herford, Germany) was used. The concentration of dog IFN-γ in the culture supernatant was evaluated by Duoset ELISA canine IFN-γ (R&D systems, Minneapolis, MN, USA) according to the manufacturer’s protocol. Absorbance was measured using a microplate reader MTP-650FA (Corona Electric, Ibaraki, Japan).

### Statistics

In the blocking assay with anti-PD-L1 antibody, Tukey’s test was conducted among 0, 0.5, 1.0, 2.5, and 5.0 µg/mL antibody treatment groups. The Wilcoxon signed rank-sum test was conducted to compare the data from the same individuals. For both tests, *p*<0.05 was considered statistically significant.

### Nucleotide Sequence Accession Numbers

The sequences of the canine PD-1 and PD-L1 genes have been submitted to the GenBank database under accession number AB898677 (PD-1) and AB898678 (PD-L1).

## Results

### Sequence Analysis of Canine PD-1 and PD-L1

**Figure 1:**
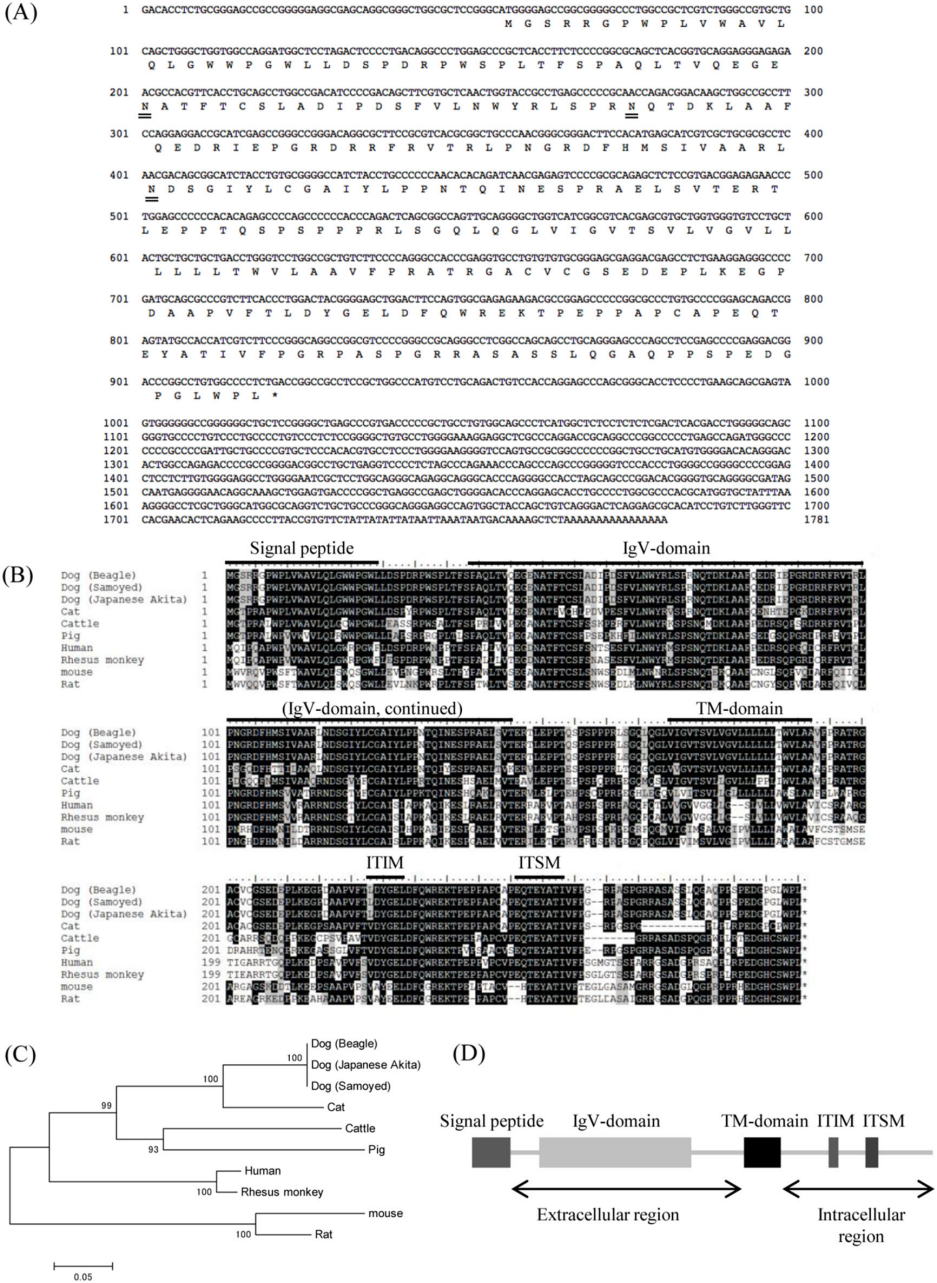
Sequence analysis of canine PD-1. (A) Nucleic acid and deduced amino acid sequences of canine PD-1 cDNA. Canine PD-1 cDNA encodes for a 288 amino acid polypeptide. Predicted N-glycosylation sites in the amino acid sequence of canine PD-1 are doubly underlined. (B) Multiple sequence alignment of vertebrate PD-1 amino acid sequences. Predicted domains and motifs of canine PD-1 are shown in the figure. Signal peptide, 1–24; IgV domain, 39–145; transmembrane domain, 170–192; ITIM, 223–228; ITSM, 246–253. (C) Phylogenetic tree of the canine PD-1 sequence in relation to those of other vertebrate species. The bootstrap consensus tree was inferred from 1000 replicates (the numbers next to the branches indicate the bootstrap percentage). The scale indicates the divergence time. (D) Schematic image of predicted functional motifs in canine PD-1. Canine PD-1 consists of an extracellular region, a transmembrane region, and an intracellular region.

**Figure 2:**
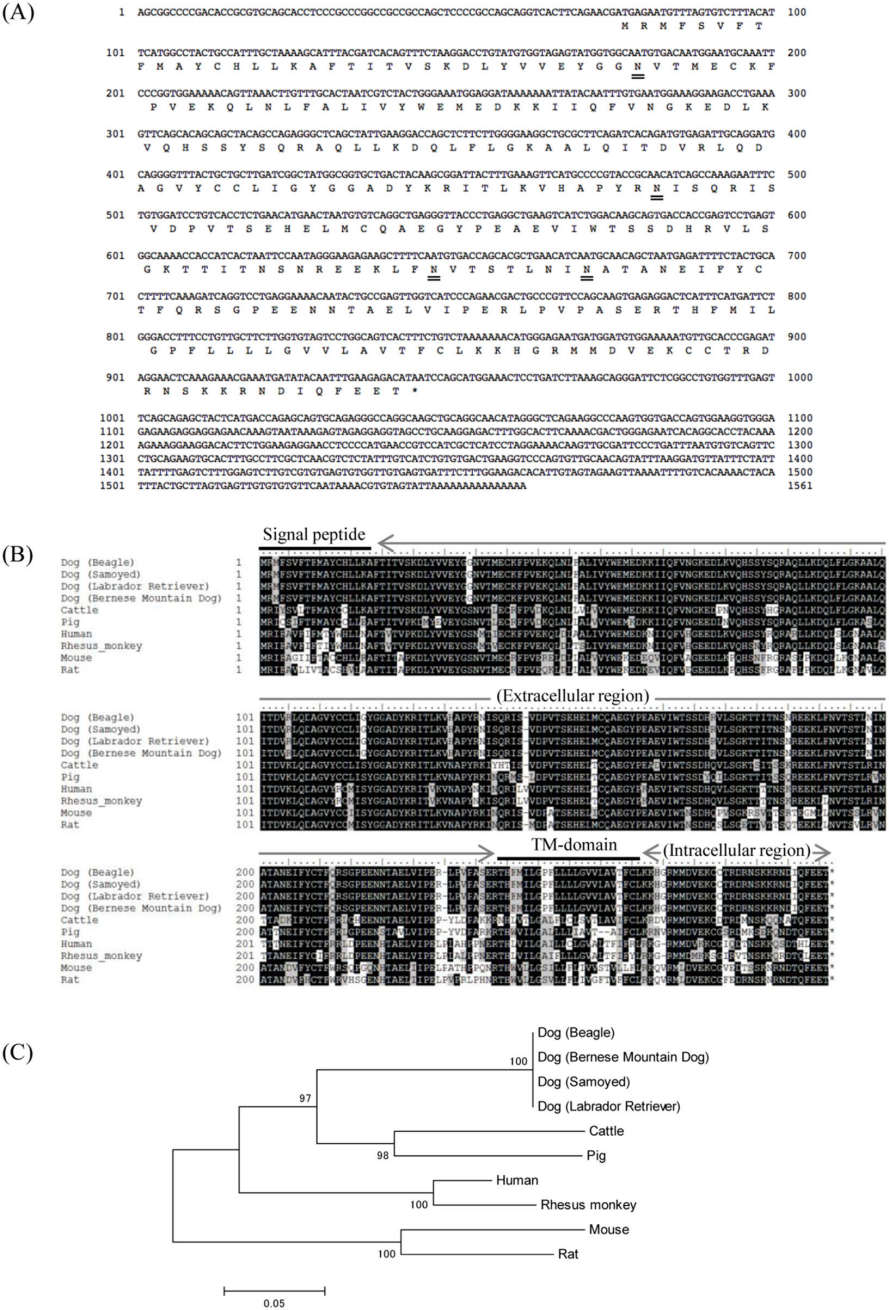
Sequence analysis of canine PD-L1. (A) Nucleic acid and deduced amino acid sequences of canine PD-L1 cDNA. Canine PD-L1 cDNA encodes for a 289 amino acid polypeptide. Predicted N-glycosylation sites in the amino acid sequence of canine PD-L1 are doubly underlined. (B) Multiple sequence alignment of vertebrate PD-L1 amino acid sequences. Predicted domains and regions of canine PD-L1 are shown in the figure. Signal peptide, 1–18; transmembrane domain, 237–259. Canine PD-L1 consists of an extracellular region, a transmembrane region, and an intracellular region. (C) Phylogenetic tree of the canine PD-L1 sequence in relation to those of other vertebrate species. The bootstrap consensus tree was inferred from 1000 replicates (the numbers next to the branches indicate the bootstrap percentage). The scale indicates the divergence time.

The complete nucleotide sequences and putative amino acid sequences of canine PD-1 and PD-L1 obtained from the Beagle dog are shown in Fig. 1A and Fig. 2A, respectively. The complete nucleotide sequences for canine PD-1 and PD-L1 were found to be 1,781 bp and 1,561 bp in length, and the sequences had an ORF encoding for 288 and 289 amino acids, respectively. Canine PD-1 and PD-L1 consist of a putative signal peptide, an extracellular domain, a transmembrane domain, and an intracellular domain as in other species (Fig. 1B, 1D and Fig. 2B). The cytoplasmic tail of PD-1 contains two structural motifs, an immunoreceptor tyrosine-based inhibitory motif (ITIM: I/L/V/S/TxYxxL/V/I), and an immunoreceptor tyrosine-based switch motif (ITSM) [38]. ITIM and ITSM were conserved in canine PD-1 (Fig. 1B, 1D). Both the phylogenetic analyses revealed that mammalian PD-1 and PD-L1 were divided into two groups, group 1 (Perissodactyla, Artiodactyla, and Carnivora) and group 2 (Primate and Rodentia), with canine PD-1 and PD-L1 clustering in the Carnivora species group (Fig. 1C and Fig. 2C). Comparative analysis of the PD-1 sequences of several species showed that canine PD-1 had 87.8%, 77.1%, 75.7%, and 68.4% amino acid similarity with cat, cow, human, and mouse, respectively (Table 1). On the other hand, PD-L1 sequences of several species showed that canine PD-L1 had 87.9%, 86.2%, and 82.4% amino acid similarity with cow, human, and mouse, respectively (Table 2). To compare the genetic diversity of the canine PD-1 and PD-L1 among other canine breeds, the samples from the Samoyed, Labrador retriever, Japanese Akita, and Bernese mountain dog were collected and compared to that of the Beagle dog. Although some sequences were not obtained because the samples did not express the genes in the tissue, their genetic sequences showed 100% nucleotide identity with the sequence from the Beagle dog (Fig. 1B and Fig. 2B).

**Table 1:** Nucleotide and amino acid sequence similarities of PD-1 among vertebrate species.

Species (GenBank accession number)	Dog	Cat	Cattle	Pig	Human	Rhesus monkey	Mouse	Rat
Dog	-	83.4	78.1	78.5	76.4	77.3	69.2	69.0
Cat (NM_001145510)	87.8	-	76.7	74.5	72.1	72.5	65.3	64.7
Cattle (AB510901)	77.1	76.6	-	80.0	74.9	75.1	66.6	67.6
Pig (NM_001204379)	76.4	75.7	79.9	-	76.8	77.0	69.8	71.2
Human (NM_005018)	75.7	73.3	72.9	71.9	-	95.8	71.4	72.0
Rhesus monkey (NM_001114358)	75.7	73.6	73.6	72.6	98.3	-	71.3	71.9
Mouse (NM_008798)	68.4	64.2	65.6	67.4	71.5	72.6	-	91.3
Rat (NM_001106927)	69.4	65.9	68.3	70.1	71.2	72.2	90.3	-

Upper section; similarities in nucleotide level, Lower section; similarities in amino acid level. Genbank accession numbers are shown in the Table.

**Table 2:** Nucleotide and amino acid sequence similarities of PD-L1 among vertebrate species.

Species (GenBank accession number)	Dog	Cattle	Pig	Human	Rhesus monkey	Mouse	Rat
Dog	-	83.3	84.6	83.2	82.4	73.2	73.6
Cattle (NM_001163412)	87.9	-	87.4	83.0	81.7	73.3	74.4
Pig (NM_001025221)	89.3	92.0	-	84.4	83.2	74.2	75.2
Human (AK314567)	86.2	85.2	86.9	-	95.7	76.3	76.5
Rhesus monkey (EF444816)	85.2	83.4	85.2	96.2	-	75.2	75.1
Mouse (AF317088)	82.4	80.7	82.4	82.8	82.1	-	87.1
Rat (NM_001191954)	82.8	80.3	82.1	82.8	82.1	92.1	-

Upper section; similarities in nucleotide level, Lower section; similarities in amino acid level. Genbank accession numbers are shown in the Table.

### Canine PD-1–Ig Binds to PD-L1, and Its Binding is Disturbed by Bovine PD-L1 Antibody

**Figure 3:**
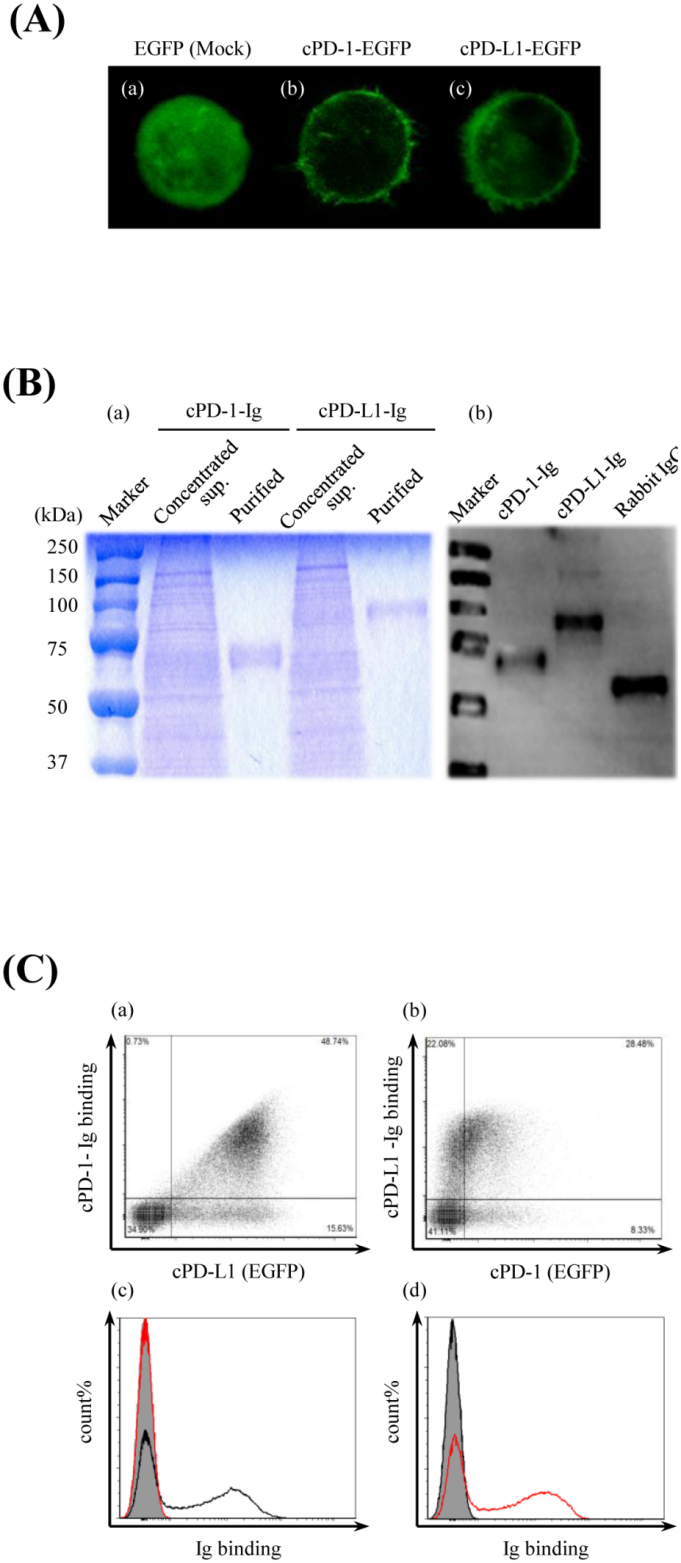
Establishment of canine PD-1– or PD-L1– expressing cells and Ig fusion recombinant proteins. (A) Canine PD-1–EGFP– or canine PD-L1–EGFP–expressing cell. The subcellular distributions of EGFP only, cPD-1–EGFP, and cPD-L1–EGFP in transiently transfected Cos7 cells were analyzed by a confocal microscope (400×). Cos7 cells were transfected with (a) pEGFP-N2 vector only (Mock), (b) pEGFP-N2–cPD-1 or (c) pEGFP-N2–cPD-L1. (B) Production and purification of Ig fusion recombinant proteins. The canine PD-1 and canine PD-L1 extracellular regions combined to the rabbit IgG Fc region (cPD-1–Ig, cPD-L1–Ig) were expressed as soluble proteins in the culture supernatant by stably expressing CHO-DG44 cells, which had been transfected with pCXN2.1–rabbit IgG Fc-cPD-1 or pCXN2.1–rabbit IgG Fc-cPD-L1. (a) SDS–PAGE analysis of the concentrated culture supernatant of the expressing cells and the purified Ig fusion proteins. (b) Western blot analysis of the purified Ig fusion proteins. Rabbit IgG was used as a positive control. (C) Canine PD-L1 binds to canine PD-1. Transiently transfected cPD-1–EGFP– or cPD-L1–EGFP–expressing Cos7 cells were incubated with cPD-L1–Ig or cPD-1–Ig, respectively. The cells were washed and the binding of the Ig fusion proteins was analyzed by flow cytometry using a fluorescent labeled anti-rabbit IgG Fc antibody. cPD-1–EGFP or cPD-L1–EGFP expression on the transfected Cos7 cells were confirmed by EGFP fluorescence. (a) Binding of cPD-1–Ig to cPD-L1–expressing cells. (b) Binding of cPD-L1–Ig to cPD-1–expressing cells. (c) Histogram analysis of cPD-1–Ig binding to cPD-L1–EGFP–expressing cells. Black line, cPD-1–Ig; red line, cPD-L1–Ig; shaded area, rabbit IgG. (d) Histogram analysis of cPD-L1–Ig binding to cPD-1–EGFP–expressing cells. Black line, cPD-1–Ig; red line, cPD-L1–Ig; shaded area, rabbit IgG.

First, to confirm the binding of canine PD-L1 to canine PD-1, we established an *in vitro* model by transfecting Cos7 cells with canine PD-1 and PD-L1 and generated transient transfectants. Expression of canine PD-1 and PD-L1 was detected on cPD-1–EGFP and cPD-L1–EGFP transfectants (Fig. 3A), respectively. On the other hand, canine PD-1–Ig and PD-L1–Ig were expressed *in vitro* using the pCXN2.1 expression vector and CHO-DG44 cell expression system. Affinity-purified canine PD-1–Ig and PD-L1–Ig migrated as molecular weights of around 65 kDa and 85 kDa protein on a 12% polyacrylamide gel, respectively (Fig. 3B). Subsequently, the binding of canine PD-L1 and PD-1 was alternately confirmed by flow cytometry analysis. As expected, the canine PD-1–Ig was bound to canine PD-L1 on cells, while the canine PD-L1–Ig was bound to canine PD-1 on cells (Fig. 3C).

**Figure 4:**
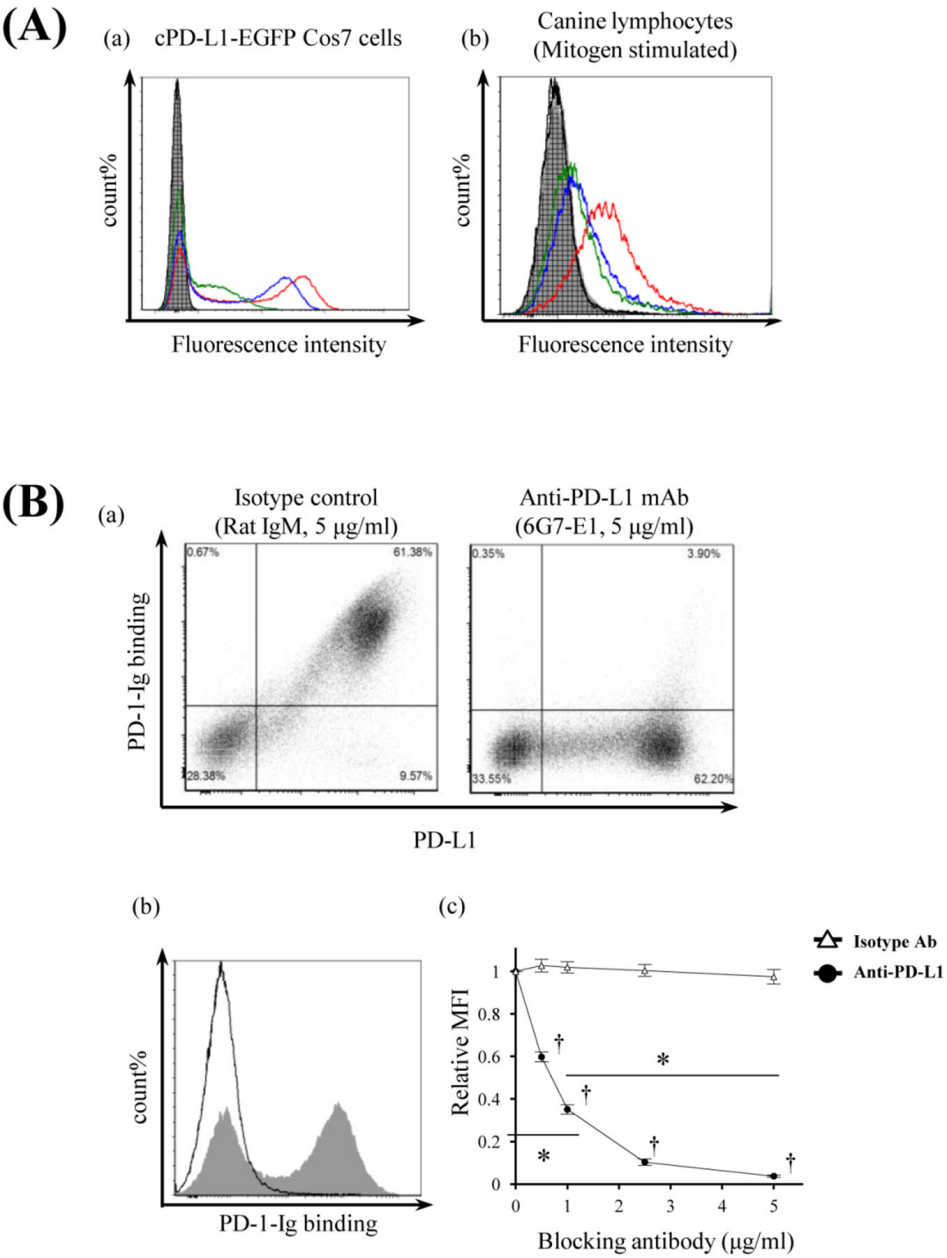
Monoclonal antibodies which recognize canine PD-L1. (A) Cross-reactivities of antibovine PD-L1 monoclonal antibodies. Binding abilities of recently established anti-boPD-L1 monoclonal antibodies to canine PD-L1 were examined by flow cytometry. Three anti-boPD-L1 monoclonal antibody clones, 4G12-C1 (rat IgG2a), 5A2-A1 (rat IgG1), and 6G7-E1 (rat IgM), were tested and all the three clones were found to recognize canine PD-L1. Rat IgG2a, rat IgG1, and rat IgM were used as isotype-matched negative controls. (a) cPD-L1–EGFP–expressing Cos7 cells and (b) dog PBMCs stimulated with PMA/ionomysin for 3 days were stained with anti-boPD-L1 monoclonal antibodies (10 µg/mL) or isotype-matched control antibodies. Red line, 4G12-C1; blue line, 5A2-A1; green line, 6G7-E1; shaded area, rat IgG2a; vertical-striped area, rat IgG1; horizontal-striped area, rat IgM. (B) Blockade of cPD-1/cPD-L1 binding by anti-PD-L1 monoclonal antibody 6G7-E1. cPD-L1–EGFP–expressing cells were preincubated with anti-PD-L1 antibody and then cPD-1–Ig bindings were evaluated by flow cytometry. (a) Blocking effect of anti-PD-L1 monoclonal antibody 6G7-E1 on cPD-1/cPD-L1 binding. Five microgram per milliliter of isotype-matched control antibody (rat IgM) could not affect the cPD-1/cPD-L1 binding (left panel), whereas the same concentration of 6G7-E1 significantly blocked the Ig binding (right panel). (b) Representative histogram of the flow cytometric analysis. Shaded area, isotype control (5 µg/mL); solid line, anti-PD-L1 monocolonal antibody 6G7-E1 (5 µg/mL). (c) Dose-dependent blocking effect of 6G7-E1 on cPD-1/cPD-L1 binding. Cells were preincubated with 6G7-E1 or isotype control antibody at various concentrations (0.5, 1.0, 2.5, 5.0 µg/mL) and Ig binding was analyzed by flow cytometry. Each point indicates the average value of relative MFI obtained from three independent experiments (compared to no antibody control, error bar; SEM). Statistical significance was evaluated by Tukey’s test (*p<0.05, between the 0 µg/mL and the 1 µg/mL of anti-PD-L1 antibody treatment group and between the 1 µg/mL and the 5 µg/mL of anti-PD-L1 group. †*p*<0.05, between the each concentration of anti-PD-L1 group and the same concentration of isotype control group).

For functional analysis of canine PD-1/PD-L1, we confirmed the cross-reactivity of bovine PD-L1 antibodies against canine PD-L1. The bovine PD-L1 antibodies 4G12-C1, 5A2-A1, and 6G7-E1 recognized the canine PD-L1 on Cos7 cells (Fig. 4A(a)) and the mitogen-stimulated canine PBMCs (Fig. 4A(b)). Furthermore, to confirm whether the PD-L1 antibody can block the binding of canine PD-L1 to PD-1, PD-L1 antibody (6G7-E1) was added in the canine PD-L1 expressing Cos7 cells, and then canine PD-1–Ig was added. PD-L1 antibody clearly inhibited the binding of canine PD-1–Ig to membrane PD-L1 in a dose-dependent manner (Fig. 4B). Five microgram per milliliter of antibody completely inhibited the binding; however, the same dose of control antibody could not inhibit the binding (Fig. 4B(c)).

### PD-L1 Expression on Canine Tumors

**Figure 5:**
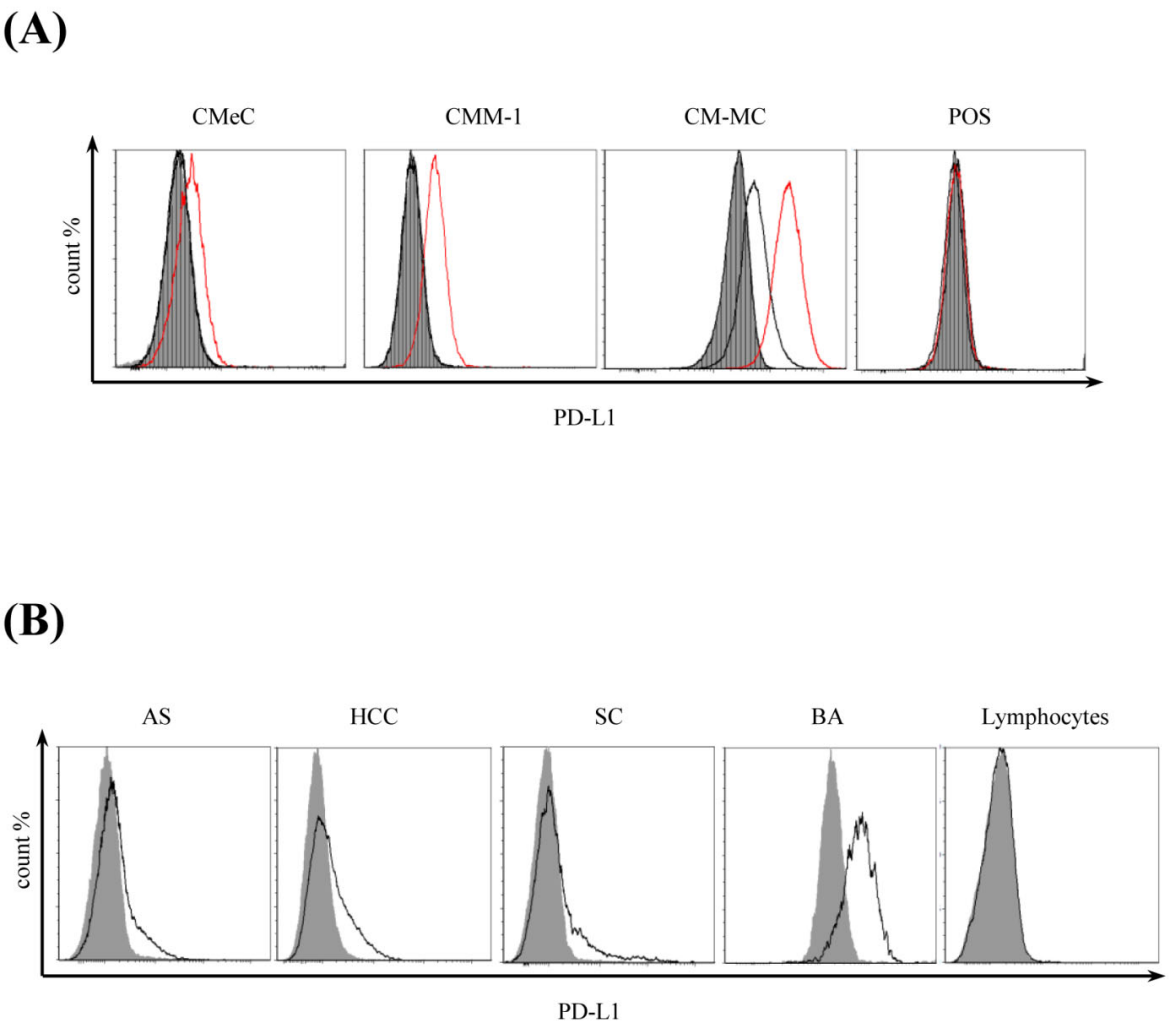
Expression of PD-L1 on dog tumor cells. (A) Representative data for the analysis of PD-L1 expression on dog tumor cell lines (Table 3). Cells maintained in the medium or those stimulated by IFN-γ (100 ng/mL) for 24 h were stained with anti-PD-L1 monoclonal antibody 4G12-C1 or isotype control antibody (rat IgG2a). Black line, medium/4G12-C1; red line, IFN-γ/4G12-C1; shaded area, medium/isotype control; vertical-striped area, IFN-γ/isotype control. (B) Tumor tissues excised surgically from clinical cases of dog tumors were treated with collagenase, and a tumor single cell suspension was obtained. To reduce the effect of collagenase on the degradation of PD-L1 and to restore the cell surface PD-L1, the tumor cells were cultured in the medium for 24 h before FACS analysis. Lymphocytes obtained from healthy dogs were used as a negative control to confirm that the collagenase and culture treatment would not upregulate the PD-L1 expression. The histogram shows the expression of PD-L1 on each tumor cell. Solid line, 4G12-C1; shaded area, isotype control. AS, angiosarcoma; HCC, hepatocellular carcinoma; SC, squamous carcinoma; and BA, breast adenocarcinoma. Details of each tumor samples are shown in Table 4.

**Figure 6:**
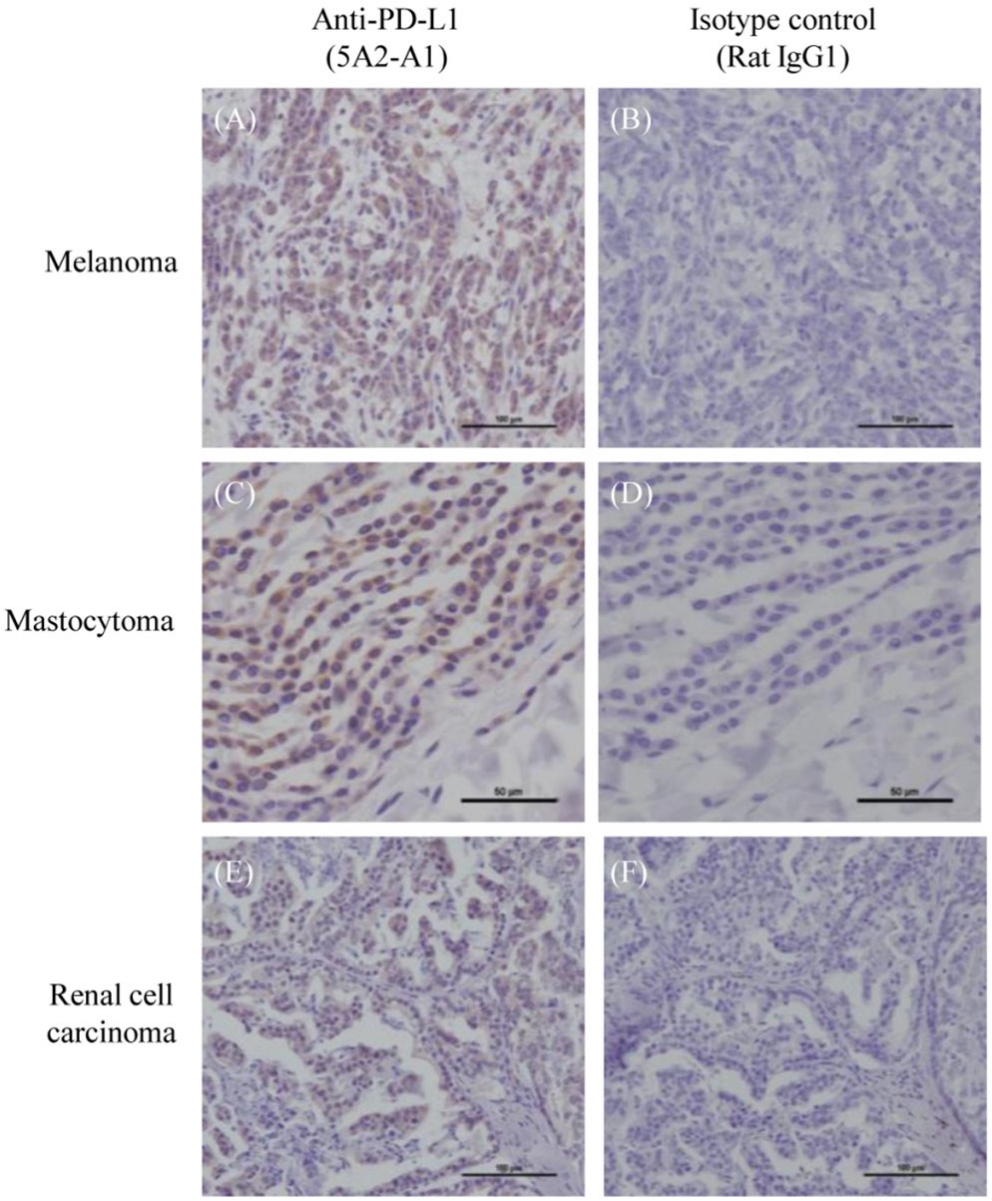
PD-L1 expression on dog tumor tissues. Immunohistochemical analysis was performed using anti-PD-L1 monoclonal antibody 5A2-A1 or isotype control antibody (rat IgG1). (A–B) Representative immunohistochemical staining of melanoma. (C–D) Representative immunohistochemical staining of mastocytoma. (E–F) Representative immunohistochemical staining of renal cell carcinoma.

First, we investigated the expression of PD-L1 on canine tumor cell lines. PD-L1 was detected on two cell lines (CM-MC, CoMS) from canine mastocytoma, and the expression was enhanced by IFN-γ treatment (Fig. 5A, Table 3). Interestingly, IFN-γ induced PD-L1 expression on all tested melanoma cell lines (CMeC, LMeC, CMM-1, and CMM-2) but not on osteosarcoma cell lines (POS, HM-POS) (Fig. 5A, Table 3). Subsequently, using clinical materials from dogs with tumors, we investigated the expression of PD-L1 by flow cytometry and identified PD-L1 expression on cells from angiosarcoma, hepatocellular carcinoma, squamous carcinoma, and breast adenocarcinoma; PD-L1 was not detectable on the lymphocytes from control dogs (Fig. 5B, Table 4). Finally, PD-L1 expression was histologically analyzed. PD-L1 was expressed in most melanoma (69.2%), mastocytoma (66.7%), and renal cell carcinoma (70.0%) cases (Fig. 6, Table 5). Although the number of tested cases was limited, PD-L1 was expressed in all oral melanoma cases (Table 5).

**Table 3:** Expressions of PD-L1 on dog tumor cell lines.

Cell line	Pathology	PD-L1 expression	
		Medium	IFN-γ*
CMeC	Melanoma	-	+
LMeC	Melanoma	-	+
CMM-1	Melanoma	-	++
CMM-2	Melanoma	-	++
CM-MC	Mastocytoma	+	+++
CoMS	Mastocytoma	+	+++
POS	Osteosarcoma	-	-
HM-POS	Osteosarcoma	-	-

The expression of PD-L1 was evaluated by flow cytometric analysis using anti-PD-L1 mAb 4G12-C1. -; <3% positive, +; 3–30% positive, ++; 30–60% positive, +++; >60% positive.

*Cells were incubated with recombinant canine IFN-γ (100 ng/ml) for 24 h before the analysis.

**Table 4:** Tumor samples used in the flow cytometric analysis of PD-L1 expression.

Pathology	PD-L1	Site	Breed	Age
Angiosarcoma	+	Thorax	Scottish terrier	11
Hepatocellular carcinoma	+	Liver	Siberian husky	13
Squamous carcinoma	+	Forefoot	Shih-tzu	14
Breast adenocarcinoma	+	Mammary gland	Hokkaido	14

Tumor tissues surgically excised from clinical cases of dog tumors were used for the flow cytometric analysis. Pathology, PD-L1 expression, Tumor site, Breed of dog, and Age of each tumor sample are shown in the table.

**Table 5:** Immunohistochemical analysis of PD-L1 expression on dog tumor tissues.

Pathology		Positive case/Tested samples (% positive)	
Melanoma	Oral cavity	8/8	(100%)
	Skin	1/3	(33.3%)
	Eye	0/2	(0%)
	All	9/13	(69.2%)
Mastocytoma		4/6	(66.7%)
Renal cell carcinoma		7/10	(70.0%)

Immunohistochemical analysis of dog PD-L1 was conducted using dog tumor tissues surgically excised from clinical cases by anti-PD-L1 mAb 5A2-A1. Melanoma cases were divided into 3 groups dependent on the tumor site (Oral cavity, Skin, Eye).

### PD-1/PD-L1 Blockade by PD-L1 Antibody Enhances IFN-γ Production

**Figure 7:**
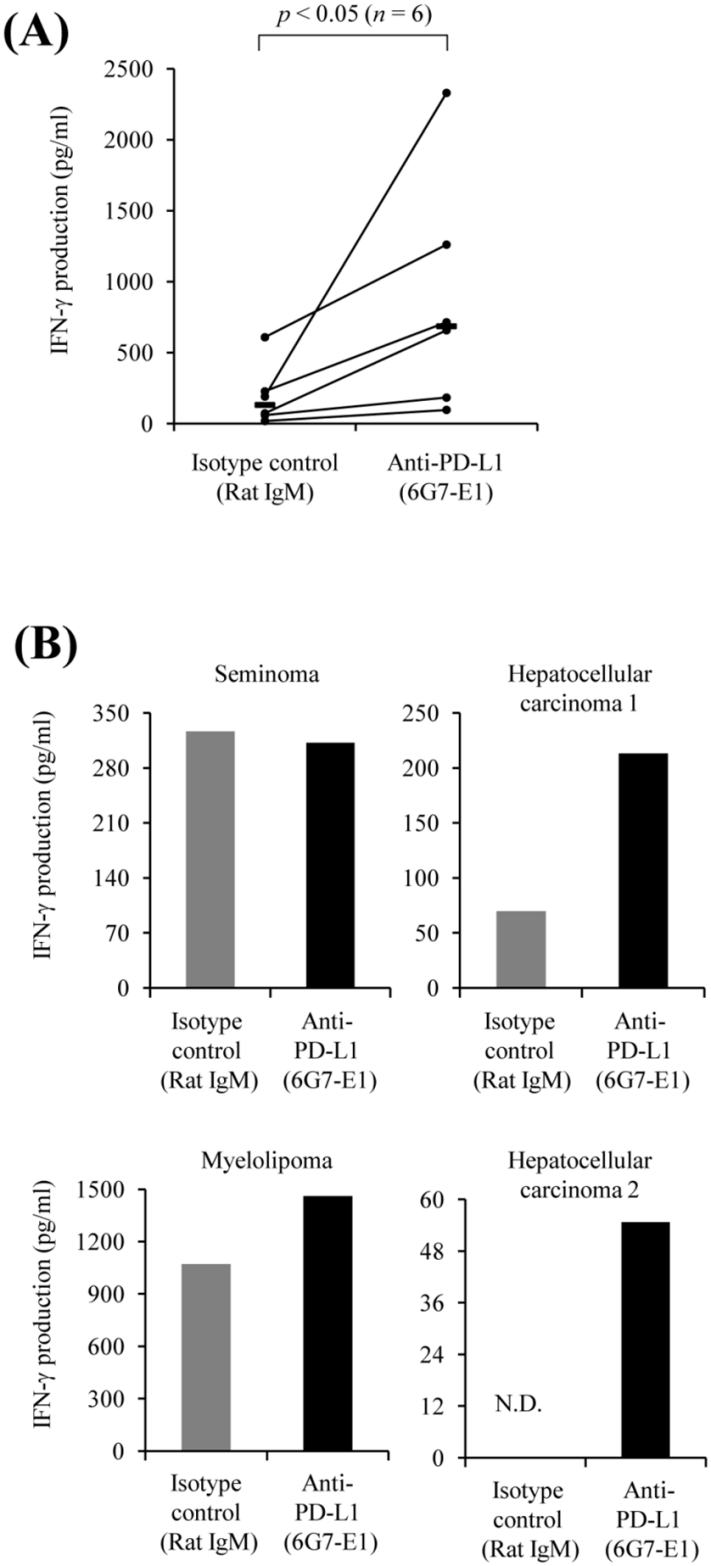
Effects of PD-L1 blockade by anti-PD-L1 monoclonal antibody. (A) Effect of anti-PD-L1 monoclonal antibody on IFN-γ production of dog PBMCs. PBMCs obtained from healthy dogs were cultured with anti-PD-L1 monoclonal antibody 6G7-E1 or isotype-matched control antibodies (20 µg/mL) for 2 days. The concentration of IFN-γ in the culture supernatant was measured by ELISA. Statistical significance was evaluated by the Wilcoxon signed rank-sum test (*n* = 6, *p*<0.05). (B) Effect of blockade of the PD-1/PD-L1 pathway on IFN-γ production of tumor-infiltrating lymphocytes. Tumor-infiltrating lymphocytes were obtained from several dog tumor tissues and cultured with 20 µg/mL of anti-PD-L1 monoclonal antibody 6G7-E1 or isotype-matched control antibody for 2 days. Details of each tumor sample are shown in Table 6. N.D., Not Detected.

To investigate the effect of PD-1/PD-L1 blockade by PD-L1 antibody in cytokine production, PBMCs from healthy dogs were cultivated in the presence of PD-L1 antibody or isotype control antibody. As shown in Fig. 7A, addition of PD-L1 antibody at the beginning of the 48 h cultivation period significantly enhanced the IFN-γ production compared with those treated with the control antibody (*p*<0.05). Finally, to confirm that PD-L1 antibody augments the production of cytokines from tumor-infiltrating cells, PD-L1 antibody was added to the infiltrating cell cultures from hepatocellular carcinoma, myelolipoma and seminoma at the beginning (Table 6). Interestingly, IFN-γ production was enhanced in the infiltrated cells from hepatocellular carcinoma and myelolipoma (Fig. 7B).

**Table 6:** Tumor samples used in the blockade test of PD-1/PD-L1 pathway.

Pathology	N-fold	Site	Breed	Age
Seminoma	0.96	Testis	Beagle	11
Hepatocellular carcinoma 1	3.06	Liver	Boston terrior	11
Myelolipoma	1.36	Spleen	Beagle	7
Hepatocellular carcinoma 2	-	Liver	Shih-tzu	9

Tumor infiltrating lymphocytes (TILs) were separated from tumor single cell suspension obtained from clinical cases and used for the blockade test. Pathology, tumor site, breed of dog, and age of each tumor sample are shown in the table. Relative value of IFN-γ production of TILs treated with anti-PD-L1 mAb compared to that of TILs treated with isotype control was also shown as N-fold in the table.

## Discussion

Several immunotherapies against tumors have been recently developed in humans. Among these therapies, an immunotherapy targeting the PD-1/PD-Ls pathway would be considered as one of the most encouraging approaches because, in a Phase I clinical trial, treatment with PD-L1 antibody induced durable tumor regression and prolonged stabilization of disease in patients with advanced malignant cancers, including non-small-cell lung cancer, melanoma, and renal cell cancer [18]. In addition to this, clinical trials targeting the PD-1/PD-L pathway by using PD-1 antibody are also ongoing for cancer therapy [19], [39]. Furthermore, several clinical trials have already been planned or are in progress, combining antibodies targeting the PD-1/PD-L pathway with cancer vaccines, antitumor antibodies, or chemotherapies in humans. However, there has been no report demonstrating that the blockade targeting the PD-1/PD-L pathway might show potential for development of new therapies against canine tumors. In the present study, to evaluate this possibility, we investigated the expression of PD-L1 in several canine tumors and found high levels of PD-L1 expression on cells from dogs with tumors. We also found that PD-1/PD-L1 blockade by PD-L1 antibody enhances IFN-γ production from tumor-infiltrating cells from dogs with tumors. These observations raise the possibility that the PD-1/PD-L1 pathway could be a therapeutic target for the treatment of dog tumors.

PD-L1 was detected on canine cell lines from mastocytoma and on fresh cells from angiosarcoma, hepatocellular carcinoma, squamous carcinoma and breast adenocarcinoma. Furthermore, the increasing expression of canine PD-L1 on tumor cells was confirmed in clinical cases of melanoma, mastocytoma, and renal cell carcinoma by immunohistochemical analysis. These findings corresponded to those of human cancers. However, the expression of PD-L1 was not observed in some cases, such as osteosarcoma-derived cell lines and tumor tissues from clinical cases, melanoma, mastocytoma, and renal cell carcinoma. PD-L1 expression is induced by cytokines such as IFNs type I and type II [40]–[42]. Indeed, the expression of PD-L1 on cell lines from canine tumors was induced and enhanced by IFN-γ treatment. According to previous studies, PD-L1 expression is associated with cytokine production within the tumor microenvironment [3]. Some cases without PD-L1 expression might be also influenced by the tumor microenvironment without cytokines. Further detailed analyses needs to be conducted to clarify the reasons for the lack of PD-L1 expression on the tumors.

In the present study, we detected significant PD-L1 expression on tumor tissues from dogs with mastocytoma and renal cell carcinoma, which originates from mast cells and renal tubular epithelial cells. Dogs have a unique risk of development of cutaneous mastocytoma, which accounts for up to 21% of all skin tumors; however, mastocytoma is rare in human and other species [43]. It is known that, in humans, PD-L1 is expressed on mast cells and it can negatively regulate several immune responses [44]. Furthermore, renal tubular epithelial cells can express PD-L1, which is involved in inhibition of proliferation and cytokine synthesis [45]. The high expression of PD-L1 on tumor cells from canine mastocytoma and renal cell carcinoma might correspond to its PD-L1–expressing original cells. Interestingly, de Haij reported that interaction of tubular epithelial cells and kidney-infiltrating T-cells via PD-L1 changed the balance of positive and negative signals to the T-cells, leading to IL-10 production and the limitation of local immune responses. These observations suggest that PD-L1–expressing cells are associated with T-cell dysfunction in the tumor microenvironment and can result in tumor formation. It will be important to clarify whether the PD-L1 expression level is related with an increased risk of disease progression. At least, canine mastocytoma and renal cell carcinoma might be candidate target cancers for immunotherapy using the PD-L1 antibody.

Melanoma is a common tumor in dogs; it is a locally invasive and frequently malignant type of cancer that can affect dogs [46], [47]. Different forms of melanoma are classified by location: skin (cutaneous melanoma), eyelids (ocular melanoma), nail bed (subungual melanoma), and oral cavity (oral melanoma). Among the various forms, canine oral melanoma is a more highly aggressive and fatal tumor [47], [48]. In addition, canine oral melanoma is frequently resistant to chemotherapy [49]–[51] and not affected by radiotherapy [52]. Thus, researchers hope for the development of novel therapy against melanoma. In this study, PD-L1 was detected in all cases of canine oral melanoma. The findings from the present study could lead to the design of novel therapeutics against canine oral melanoma, although it remains to be determined whether PD-L1 is expressed on cells from other types of melanomas.

As a preliminary test of the hypothesis that PD-L1 may be a therapeutic target for the treatment of canine tumors, we investigated the effects of blockade of PD-1/PD-L1 by PD-L1 antibody in tumor-infiltrating T-cells from dogs with seminoma, hepatocellular carcinoma and myelolipoma. Similar to previous findings in human or mice models, inhibition of the PD-1/PD-L1 pathway had upregulated the production of IFN-γ from lymphocytes from hepatocellular carcinoma and myelolipoma. However, although the effects of PD-L1 antibody on IFN-γ production seem to be profound, it still remains speculative whether the blockade of PD-1/PD-L1 by PD-L1 antibody will be sufficient for tumor regression *in vivo*. Furthermore, the level of PD-1 expression on the tumor-infiltrating T-cells is still unknown because of the lack of a specific antibody for canine PD-1. These preliminary results must therefore be investigated more in detail.

In conclusion, we here presented aberrant expression of PD-L1 on tumors in dogs and discussed their potential as therapeutic targets for canine tumors. PD-L1 might contribute to the progression of canine tumors via antitumor T-cell dysfunction. In the clinical trials targeting the PD-1/PD-L1 pathway in humans, none of the patients with PD-L1–negative tumors had a positive response; however, reactivation of the antitumor immunity and subsequent regression of some tumors were induced in patients with PD-L1–positive tumors, including malignant cases [19]. The findings indicate that detection of PD-L1 on tumor biopsies might be a powerful and effective method for prediction of prognosis after treatments. At least, it may be worth investigating the antitumor effects of an immunotherapy targeting the PD-1/PD-L1 pathway on canine tumors that were PD-L1 positive in this study. Studies are underway to evaluate the possible clinical application of the PD-L1 antibody as a novel therapy against canine tumors.
